# Design and synthesis of hybrid cyclophanes containing thiophene and indole units via Grignard reaction, Fischer indolization and ring-closing metathesis as key steps

**DOI:** 10.3762/bjoc.11.165

**Published:** 2015-08-31

**Authors:** Sambasivarao Kotha, Ajay Kumar Chinnam, Mukesh Eknathrao Shirbhate

**Affiliations:** 1 Department of Chemistry, Indian Institute of Technology-Bombay, Powai, Mumbai-400 076, India, Fax: 022-2572 7152

**Keywords:** cyclophane, Grignard reaction, Fischer indolization, ring-closing metathesis

## Abstract

We demonstrate a new synthetic strategy to cyclophanes containing thiophene and indole moieties via Grignard addition, Fischer indolization and ring-closing metathesis as key steps.

## Introduction

Modern olefin metathesis catalysts enable a late stage ring-closing step starting with bisolefinic substrates containing polar functional groups [1]. As part of a major program aimed at developing new and intricate strategies to cyclophanes [2–10], we envisioned various building blocks [11] by ring-closing metathesis (RCM) as a key step [12–25]. Cyclophanes containing different heterocyclic systems are difficult to assemble [26–31]. However, we believe that architecturally complex cyclophanes can be accessed by employing a reasonable selection of a synthetic strategy [32]. To enhance the chemical space and also the diversity of cyclophanes the development of powerful and general synthetic methods is highly desirable. Herein, we report a new approach to thiophene- and indole-containing hybrid cyclophane derivatives via Grignard addition, Fischer indolization and RCM as key steps.

## Strategy

**Figure 1:**
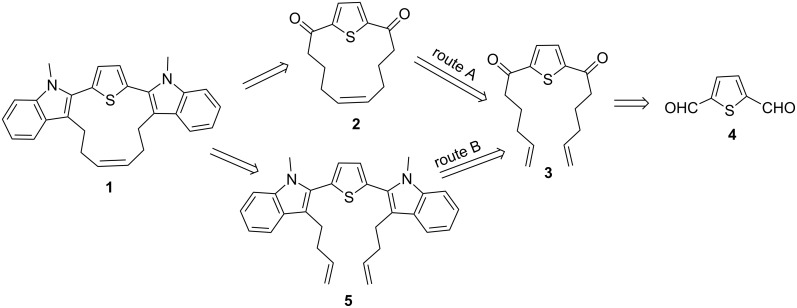
Retrosynthetic approach to hybrid cyclophane derivative **1**.

The retrosynthetic strategy to the target cyclophane **1** containing the thiophene and indole moieties is shown in Figure 1. Here, we conceived thiophene-containing diolefin **3** as a possible synthon to assemble the target molecule **1** via **2**. Route A involves an RCM of **3** followed by Fischer indolization of **2** (Figure 1). Alternatively, Fischer indolization of **3** followed by an RCM of diindole **5** can deliver target molecule **1** (Route B). The advantages of these approaches are: one can vary the length of the alkene chain during the Grignard addition, and generate diverse cyclophanes of different ring size. Diverse aromatic rings can be incorporated by altering the aryl hydrazones during the Fischer indolization step. Finally, the additional double bond generated during the RCM sequence can be further manipulated synthetically.

## Results and Discussions

**Scheme 1:**
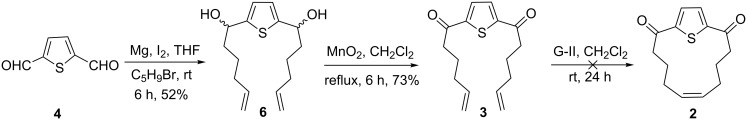
Attempted synthesis of thiophenophane derivative **2**.

Our synthetic approach to the hybrid cyclophane derivative **1** containing thiophene and indole units started with a Grignard addition reaction. In this context, commercially available thiophene-2,5-dicarbaldehyde (**4**) was reacted with the Grignard reagent [23] derived from 5-bromo-1-pentene to give diol **6** as a diastereomeric mixture (Scheme 1). Alternatively, the dialdehyde **4** can be prepared by using the Vilsmeier–Haack reaction starting with the thiophene [33]. Later, diol **6** was oxidized with MnO_2_ [34] to deliver diketone **3**. Our attempts to realize the RCM product **2** with dione **3** via a reaction with Grubbs’ catalyst failed to give the expected cyclized product. In most instances, we observed the degradation of the starting material leading to a complex mixture of products as indicated by thin-layer chromatography (TLC). It is known that sulfur can coordinate with the ruthenium catalyst and deactivate the catalytic cycle [35–37]. Therefore, the diolefin did not undergo the RCM sequence.

**Scheme 2:**
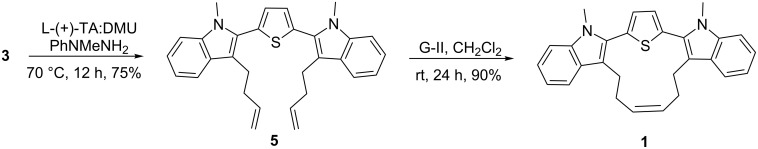
Synthesis of hybrid cyclophane **1**.

**Figure 2:**
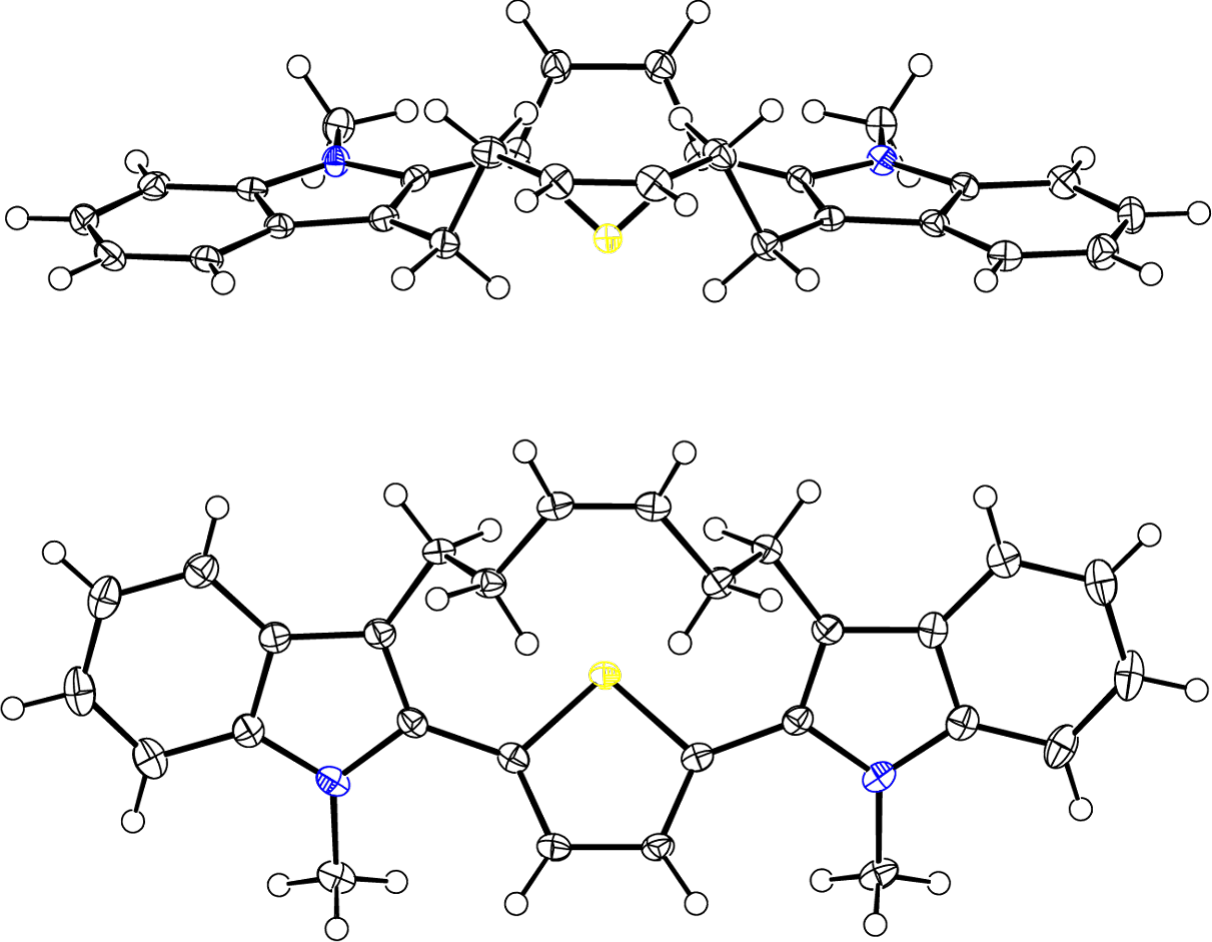
The molecular crystal structure of **1** with 50% probability [41].

Next, we explored the alternative option to the target cyclophane **1** involving the bisindolization followed by RCM (Figure 1, Route B). To design aza-polyquinanes, we reported several bisindole derivatives starting with diketones under conditions of a low melting reaction mixture [38–40]. Based on this insight, diketone **3** was subjected to a double Fischer indolization with 1-methyl-1-phenylhydrazine under conditions of a low melting reaction mixture to generate the bisindole derivative **5**. It is interesting to note that conventional conditions (AcOH/HCl) for Fischer indolization were not successful with systems related to **3**. Later, the bisindole derivative **5** was subjected to RCM in the presence of Grubbs’ 2^nd^ generation catalyst to deliver the desired product **1** in good yield (Scheme 2). The sulfur atom present in the bisolefin **3** is more accessible for coordination with the Grubbs’ catalyst. Whereas in case of the rigid bisindole the sulfur atom is somewhat shielded by the two bulky indole units. Therefore, the bisolefin **5** had undergone RCM easily. The structure of compound **1** has been assigned on the bases of ^1^H and ^13^C NMR spectra. However, the configuration of the double bond present in **1** cannot be unambiguously assigned (δ = 5.63, t, *J* = 5.40 Hz, 2H). The stereochemistry of the double bond was assigned based on single crystal X-ray diffraction studies and it was found to be the *cis* (Figure 2) [41].

**Scheme 3:**
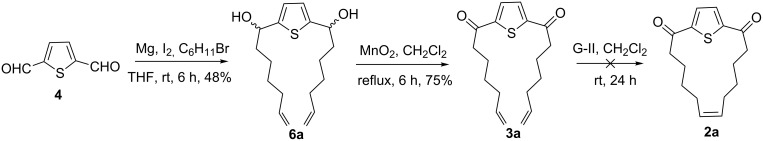
Attempted synthesis of thiophenophane derivative **2a**.

**Scheme 4:**
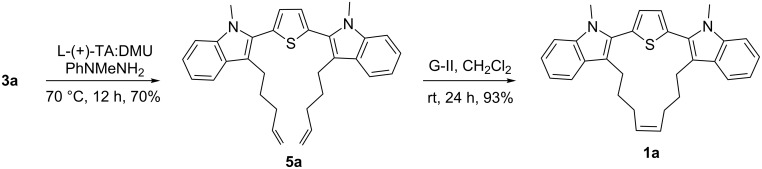
Synthesis of cyclophane **1a** with a thiophene and an indole moiety.

Having, demonstrated the RCM step, next, we attempted to expand this strategy. In this regard a synthesis of a higher analogue containing seven carbon alkenyl chains was undertaken. To achieve this goal, thiophene dicarbaldehyde **4** was subjected to a Grignard addition reaction with hexenylmagnesium bromide which gave diol **6a** as a mixture of diastereomers. Further, the diol was subjected to an oxidation step in the presence of MnO_2_ to generate dione **3a**. Later, RCM was attempted with various Grubbs’ catalysts. However, the RCM product **2a** was not realized (Scheme 3). Under similar reaction conditions, dione **3a** was converted into the bisindole derivative **5a** by using the Fischer indolization and subsequently an RCM protocol to convert **5a** to the cyclized product **1a** (Scheme 4). Based on the structure of compound **1**, here also we anticipate the double bond stereochemistry as “*cis*”.

## Conclusion

We have developed a simple synthetic strategy to hybrid cyclophane derivatives **1** and **1a** containing thiophene and indole moieties. Simple dialkene-containing thiophene derivative **3a** failed to deliver the RCM product. However, the sterically congested bisindole systems **5** and **5a** undergo RCM easily. Here, the bulky indole moieties shield the sulfur atom and prevent its coordination with the catalyst. In essence, the power of this synthetic strategy has been harnessed to realize complex cyclophanes starting with simple synthons.

## Experimental

Analytical TLC was performed on (10 × 5 cm) glass plate coated with silica gel GF_254_ (containing 13% CaSO_4_ as a binder). Visualization of the spots on the TLC plate was achieved by exposure to UV light and/or I_2_ vapor. Column chromatography was performed using silica gel (100–200 mesh) and the column was usually eluted with an ethyl acetate/petroleum ether mixture (bp 60–80 °C). Melting points were recorded on a Büchi apparatus. ^1^H NMR and ^13^C NMR spectral data were recorded on Bruker 400 and 500 MHz spectrometers using TMS as an internal standard and CDCl_3_ as solvent. The coupling constants (*J*) are given in hertz (Hz). Chemical shifts are expressed in parts per million (ppm) downfield from internal reference, tetramethylsilane. The standard abbreviation s, d, t, q, m, dd and td, refer to singlet, doublet, triplet, quartet, multiplet, doublet of doublet, and triplet of the doublet, respectively. Mass spectral data were recorded on a Q–TOF micromass spectrometer. For the preparation of anhydrous THF, initially it was passed through a column of activated alumina. Later, it was refluxed over and distilled from P_2_O_5_ and stored over sodium wire. Other reagents and solvents were purchased from commercial suppliers and used without further purification.

### General procedure for the Grignard reaction

Analogously as described in [23], Mg turnings and iodine in THF were heated to reflux until the brown colour disappeared. Then, 5-bromo-1-pentene (273 mg, 1.92 mmol) was added and the reaction mixture was stirred for 30 min. Next, thiophene 2,5-dialdehyde (**4**, 100 mg, 0.71 mmol) was added and the resulting mixture was stirred and heated at reflux for 3 h. After completion of the reaction (TLC monitoring), 2 N HCl was added and reaction mixture was stirred for 30 min. The reaction mixture was diluted with EtOAc (10 mL) and H_2_O (10 mL) and extracted with EtOAc. The organic layer was washed with brine, dried over Na_2_SO_4_, and concentrated under reduced pressure. The crude products were purified by column chromatography to obtain the diol **6** (or **6a**).

**Compound 6**: Semi solid, 104 mg (52%), by using the general procedure 100 mg (0.71 mmol) of thiophene-2,5-carbaldehyde **4** was reacted with 4-pentenylmagnesium bromide. IR (neat): 3943, 3677, 3601, 3050, 2923, 1261, 739 cm^−1^; ^1^H NMR (400 MHz, CDCl_3_) δ 1.35–1.48 (m, 2H), 1.50–1.60 (m, 2H), 1.74–1.89 (m, 4H), 2.09 (q, *J* = 7.10 Hz, 4H), 2.59 (bs, 2H), 4.81 (t, *J* = 6.50 Hz, 2H), 4.94–5.03 (m, 4H), 5.73–5.83 (m, 2H), 6.78 (s, 2H) ppm; ^13^C NMR (100.6 MHz, CDCl_3_) δ 25.15, 33.53, 38.60, 70.36, 70.39, 114.93, 123.33, 138.57, 147.99; HRMS (Q-Tof) *m*/*z*: [M + Na]^+^ calcd for C_16_H_24_NaO_2_S, 303.1389; found, 303.1394.

**Compound 6a:** Semi solid, 107 mg (48%), by using the general procedure 100 mg (0.71 mmol) of thiophene-2,5-carbaldehyde **4** was reacted with 5-hexenylmagnesium bromide. IR (neat): 743, 1270, 2933, 3042, 3589, 3694, 3942 cm^−1^; ^1^H NMR (400 MHz, CDCl_3_) δ 1.27–1.52 (m, 8H), 1.72–1.89 (m, 4H), 2.01–2.11 (m, 4H), 2.57 (bs, 2H), 4.77–4.84 (m, 2H), 4.91–5.03 (m, 4H), 5.73–5.83 (m, 2H), 6.77 (s, 2H) ppm; ^13^C NMR (100.6 MHz, CDCl_3_) δ 25.41, 28.74, 33.73, 39.03, 70.43, 114.58, 123.33, 138.89, 148.03 ppm; HRMS (Q-Tof) *m*/*z*: [M + H]^+^ calcd for C_18_H_29_O_2_S, 309.1888; found, 309.1959.

### General procedure for the MnO_2_ oxidation

To the solution of diol derivative **6** (or **6a**) (50 mg) in CH_2_Cl_2_ (10 mL) was added MnO_2_ (4 equiv) as the oxidizing agent at rt and reaction mixture was heated at reflux overnight. After completion of the reaction (TLC monitoring), the crude reaction mixture was filtered through a Celite pad (washed with CH_2_Cl_2_) and concentrated under reduced pressure. The crude product was purified by column chromatography (silica gel; 5% EtOAc/petroleum ether) to give bisalkene dione derivative **3** (or **3a**).

**Compound 3:** Semi solid, 71 mg (73%), by using the general procedure 100 mg (0.35 mmol) of thiophene derivative **6** was oxidized with MnO_2_ to deliver **3**. IR (neat): 738, 1267, 1687, 2934, 3055, 3357, 3690, 3945 cm^−1^; ^1^H NMR (400 MHz, CDCl_3_) δ 1.84 (q, *J* = 7.28 Hz, 4H), 2.15 (q, *J* = 7.05 Hz, 4H), 2.93 (t, *J* = 4.12 Hz, 4H), 4.99–5.07 (m, 4H), 5.75–5.85 (m, 2H), 7.67 (s, 2H) ppm; ^13^C NMR (100.6 MHz, CDCl_3_) δ 23.45, 33.16, 38.86, 115.77, 131.52, 137.81, 148.82, 193.55 ppm; HRMS (Q-Tof) *m*/*z*: [M + H]^+^ calcd for C_16_H_21_O_2_S, 277.1262; found, 277.1266.

**Compound 3a:** Semi solid, 74 mg (75%), by using the general procedure 100 mg (0.32 mmol) of thiophene derivative **6a** was oxidized with MnO_2_ to deliver **3a**. IR (neat): 740, 1270, 1685, 2939, 3051, 3361, 3689, 3950 cm^−1^; ^1^H NMR (400 MHz, CDCl_3_): *δ =* 1.44–1.49 (m, 4H), 1.73–1.80 (m, 4H), 2.10 (q, *J* = 7.24 Hz, 4H), 2.92 (t, *J* = 7.50 Hz, 4H), 4.95–5.05 (m, 4H), 5.75–5.85 (m, 2H), 7.67 (s, 2H) ppm; ^13^C NMR (100.6 MHz, CDCl_3_) δ 24.05, 28.59, 33.66, 39.68, 115.03, 131.55, 138.50, 148.83, 193.68 ppm; HRMS (Q-Tof) *m*/*z*: [M + H]^+^ calcd for C_18_H_25_O_2_S, 305.1574; found, 305.1557.

### General procedure for the preparation of diindole derivatives

Analogously as described in [39–40], in a typical experiment, 1.5 g of a mixture of L-(+)-tartaric acid/*N*,*N*′-dimethylurea (30:70) was heated to 70 °C to obtain a clear melt. To this melt, 2 mmol of *N*-methyl-*N*-phenylhydrazine and 1 mmol of diketone were added at 70 °C. After completion of the reaction (TLC monitoring by mini work up), the reaction mixture was quenched with water while it was still hot. The reaction mixture was cooled to rt and the solid was filtered through a sintered glass funnel and washed with water (2 × 5 mL). The crude product was dried under vacuum and then it was purified by silica gel column chromatography.

**Compound 5:** Pale yellow oil, 123 mg (75%), by using the general procedure 100 mg (0.36 mmol) of dione **3** was converted into diindole derivative **5**. IR (neat): 1048, 1097, 1242, 1374, 1447, 1465, 2927, 2974, 3019 cm^−1^; ^1^H NMR (500 MHz, CDCl_3_) δ 2.49–2.51 (m, 4H), 2.99–3.04 (m, 4H), 3.80 (s, 6H), 5.00–5.13 (m, 4H), 5.92–5.98 (m, 2H), 7.20–7.24 (m, 4H), 7.32–7.36 (m, 2H), 7.39–7.41 (m, 2H), 7.70–7.73 (m, 2H) ppm; ^13^C NMR (100.6 MHz, CDCl_3_) δ 24.86, 31.06, 35.70, 109.65, 114.83, 115.88, 119.44, 119.53, 122.59, 127.52, 129.16, 129.72, 134.45, 137.67, 138.79 ppm; HRMS (Q-Tof) *m*/*z*: [M + H]^+^ calcd for C_30_H_31_N_2_S, 451.2208; found, 451.2212.

**Compound 5a:** Pale yellow oil, 110 mg (70%), by using the general procedure 100 mg (0.33 mmol) of dione **3a** was converted into bisindole derivative **5a**. IR (neat): 738, 1267, 2934, 3055, 3357, 3690, 3945 cm^−1^; ^1^H NMR (500 MHz, CDCl_3_) δ 1.83 (t, *J* = 6.50 Hz, 4H), 2.15–2.16 (m, 4H), 2.89–2.93 (m, 4H), 3.78 (s, 6H), 4.95–5.04 (m, 4H), 5.81–5.90 (m, 2H), 7.18–7.21 (m, 4H), 7.30–7.33 (m, 2H), 7.37–7.39 (m, 2H), 7.67–7.79 (m, 2H) ppm; ^13^C NMR (125.6 MHz, CDCl_3_) δ 24.59, 30.69, 31.04, 33.91, 109.63, 114.67, 116.52, 119.48, 122.57, 127.62, 129.18, 129.67, 134.56, 137.70, 138.92 ppm; HRMS (Q-Tof) *m*/*z*: [M + H]^+^ calcd for C_32_H_35_N_2_S,479.2521; found, 479.2548.

### General procedure for RCM reaction

Analogously as described in [42], a solution of bisindole-alkene derivative **5** (0.05 mmol) in dry CH_2_Cl_2_ (50 mL) was degassed with N_2_ gas for 10 min. Then, Grubbs’ second generation catalyst (10 mol %) was added and the reaction mixture was stirred at room temperature for 24 h. After completion of the reaction (TLC monitoring), the solvent was removed under reduced pressure and the crude product was purified by silica gel column chromatography (5% EtOAc/petroleum ether) to give the RCM compound **1** as a colourless solid.

**Compound 1:** White solid, 25 mg (90%), by using the general procedure 30 mg (0.06 mmol) of bisindole **5** was treated with Grubbs’ second generation catalyst to deliver RCM product **1**. Mp 187–189 °C; IR (neat): 1098, 1265, 1364, 1458, 1644, 1734, 2858, 2926 cm^−1^; ^1^H NMR (400 MHz, CDCl_3_) δ 2.09–2.15 (m, 4H), 2.96–3.01 (m, 4H), 3.92 (s, 6H), 5.63 (t, *J* = 5.40 Hz, 2H), 7.15–7.19 (m, 4H), 7.28–7.30 (m, 2H), 7.37 (d, *J* = 8.16 Hz, 2H), 7.65 (d, *J* = 7.88 Hz, 2H) ppm; ^13^C NMR (125.6 MHz, CDCl_3_): *δ* 26.20, 28.14, 30.80, 109.64, 115.73, 118.89, 119.65, 122.54, 127.75, 128.23, 130.16, 130.31, 134.28, 137.05 ppm; HRMS (Q-Tof) *m*/*z*: [M + H]^+^ calcd for C_28_H_27_N_2_S, 423.1895; found, 423.1893.

**Compound 1a:** White solid, 35 mg (93%), By using the general procedure 40 mg (0.08 mmol) of diindole **5a** was treated with Grubbs’ second generation catalyst to deliver RCM product **1a**. Mp 183–185 °C; IR (neat): 1048, 1245, 1374, 1448, 1742, 1889, 2085, 2943, 2987, 3464, 3628 cm^−1^; ^1^H NMR (500 MHz, CDCl_3_) δ 1.76–1.78 (m, 4H), 2.03 (d, *J* = 5.25 Hz, 4H), 2.96 (t, *J* = 7.80 Hz, 4H), 3.80 (s, 6H), 5.37 (s, 2H), 7.13–7.18 (m, 4H), 7.27–7.30 (m, 2H), 7.35–7.37 (m, 2H), 7.67 (d, *J* = 7.85 Hz, 2H) ppm; ^13^C NMR (125.6 MHz, CDCl_3_) δ 23.33, 30.45, 30.96, 31.14, 109.64, 116.79, 119.43, 122.49, 127.68, 128.95, 129.68, 130.52, 134.11, 137.58 ppm; HRMS (Q-Tof) *m*/*z*: [M + H]^+^ calcd for C_30_H_31_N_2_S, 451.2208; found, 451.2192.

## Supporting Information

File 1 Copies of ^1^H, ^13^C NMR and HRMS spectra for all new compounds.

## References

[1] Nilewski C, Carreira E M (2012). Eur J Org Chem.

[2] Hopf H, Gleiter R (2004). Modern Cyclophane Chemistry.

[3] Keehn P M, Rosenfeld S M (1983). Cyclophanes.

[4] Pigge F C, Ghasedi F, Rath N P (2002). J Org Chem.

[5] Gibe R, Green J R, Davidson G (2003). Org Lett.

[6] Reiser O, König K, Meerholz K, Heinze J, Wellauer T, Gerson F, Frim R, Rabinovitz M, de Meijere A (1993). J Am Chem Soc.

[7] Fallis A G (2004). Synthesis.

[8] Frampton M J, Anderson H L (2007). Angew Chem, Int Ed.

[9] Xi H-T, Zhao T, Sun X-Q, Miao C-B, Zong T, Meng Q (2013). RSC Adv.

[10] Wex B, Jradi F M, Patra D, Kaafarani B R (2010). Tetrahedron.

[11] Kotha S (2003). Acc Chem Res.

[12] Fürstner A, Stelzer F, Rumbo A, Krause H (2002). Chem – Eur J.

[13] Huang M, Song L, Liu B (2010). Org Lett.

[14] Kotha S, Chavan A S, Shaikh M (2012). J Org Chem.

[15] Alcaide B, Almendros P, Quirós M T, Lázaro C, Torres M R (2014). J Org Chem.

[16] Kotha S, Mandal K (2009). Chem – Asian J.

[17] Kotha S, Dipak M K (2012). Tetrahedron.

[18] Kotha S, Sreenivasachary N, Mohanraja K, Durani S (2001). Bioorg Med Chem Lett.

[19] Kotha S, Sreenivasachary N (1998). Bioorg Med Chem Lett.

[20] Kotha S, Bansal D, Singh K, Banerjee S (2011). J Organomet Chem.

[21] Kotha S, Manivannan E (2003). ARKIVOC.

[22] Kotha S, Ali R, Chinnam A K (2014). Tetrahedron Lett.

[23] Kotha S, Waghule G T, Shirbhate M E (2014). Eur J Org Chem.

[24] Kotha S, Shirbhate M E (2014). Tetrahedron Lett.

[25] Kotha S, Waghule G T (2014). Tetrahedron Lett.

[26] Raatikainen K, Huuskonen J, Kolehmainen E, Rissanen K (2008). Chem – Eur J.

[27] Rajakumar P, Swaroop M G (2006). Tetrahedron Lett.

[28] Dohm J, Vögtle F (1992). Top Curr Chem.

[29] Matsuoka Y, Ishida Y, Sasaki D, Saigo K (2008). Chem – Eur J.

[30] Tanaka K (2007). Synlett.

[31] Garrison J C, Panzner M J, Tessier C A, Youngs W J (2005). Synlett.

[32] Deslongchamps P (1991). Aldrichimica Acta.

[33] Mikhaleva A I, Ivanov A V, Skital’tseva E V, Ushakov I A, Vasil’tsov A M, Trofimov B A (2009). Synthesis.

[34] Wei X, Taylor R J K (2000). J Org Chem.

[35] Shon Y-S, Lee T R (1997). Tetrahedron Lett.

[36] Ghosh S, Ghosh S, Sarkar N (2006). J Chem Sci.

[37] Samojłowicz C, Grela K (2011). ARKIVOC.

[38] Gore S, Baskaran S, König B (2012). Org Lett.

[39] Kotha S, Chinnam A K (2014). Synthesis.

[40] Kotha S, Chinnam A K (2015). Heterocycles.

[R41] 41CCDC 1060941 contains the supplementary crystallographic data for this paper. These data can be obtained free of charge from The Cambridge Crystallographic Data Centre via http://www.ccdc.cam.ac.uk

[42] Kotha S, Chavan A S, Dipak M K (2011). Tetrahedron.

