# Supplementary material for: Design and synthesis of hybrid cyclophanes containing thiophene and indole units via Grignard reaction, Fischer indolization and ring-closing metathesis as key steps
Source: Beilstein J Org Chem. 2015 Aug 31;11:1514–9. doi: 10.3762/bjoc.11.165 (PMC4578400; doi:10.3762/bjoc.11.165)

Supporting Information

for

Design and synthesis of hybrid cyclophanes containing thiophene and indole units via Grignard reaction, Fischer indolization and ring-closing metathesis as key steps

Sambasivarao Kotha,* Ajay Kumar Chinnam§ and Mukesh E. Shirbhate§

Address: Department of Chemistry, Indian Institute of Technology-Bombay, Powai, India,

Fax: 022-2572 7152

Email: Sambasivarao Kotha - srk@chem.iitb.ac.in

*Corresponding author

§A.K.C. and M.E.S. made an equal contribution to the paper.

Copies of ^{1}H, ^{13}C NMR and HRMS spectra for all new compounds

^{1}H NMR (400 MHz, $CDCl_3$) and ^{13}C NMR (100 MHz) of compound 6

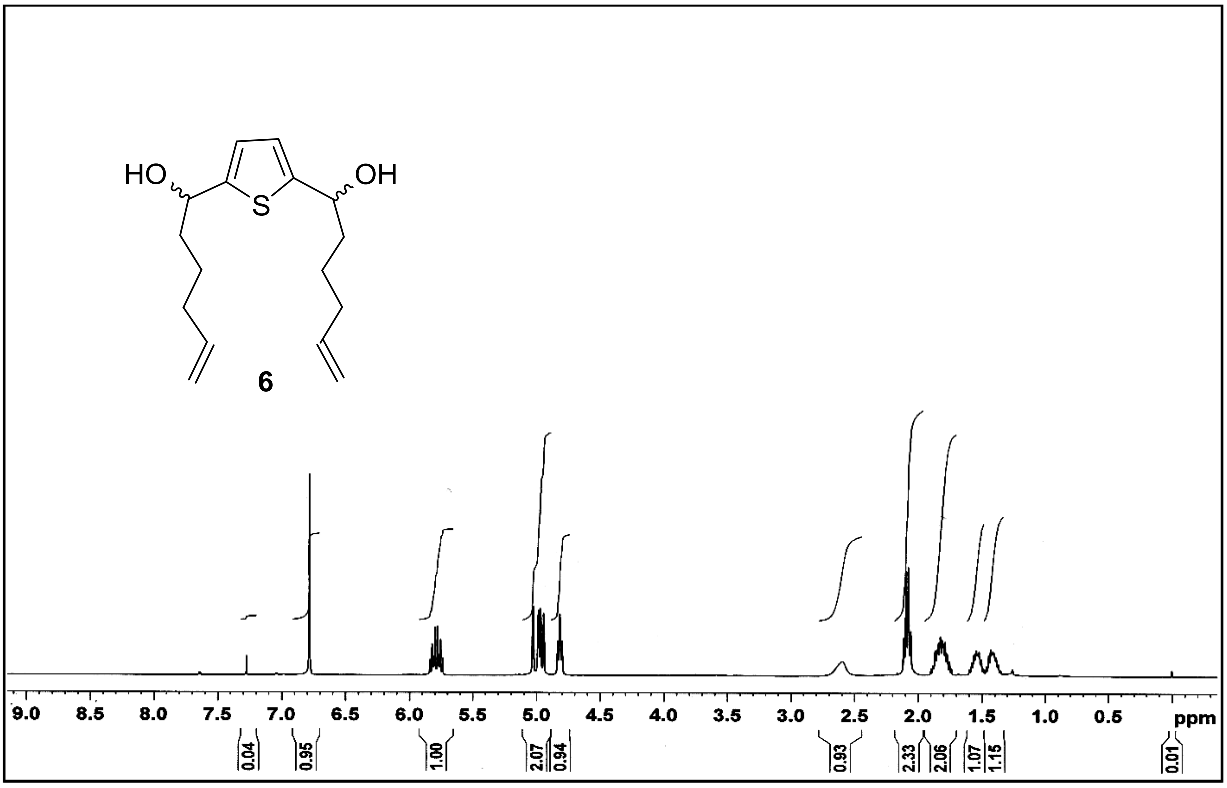

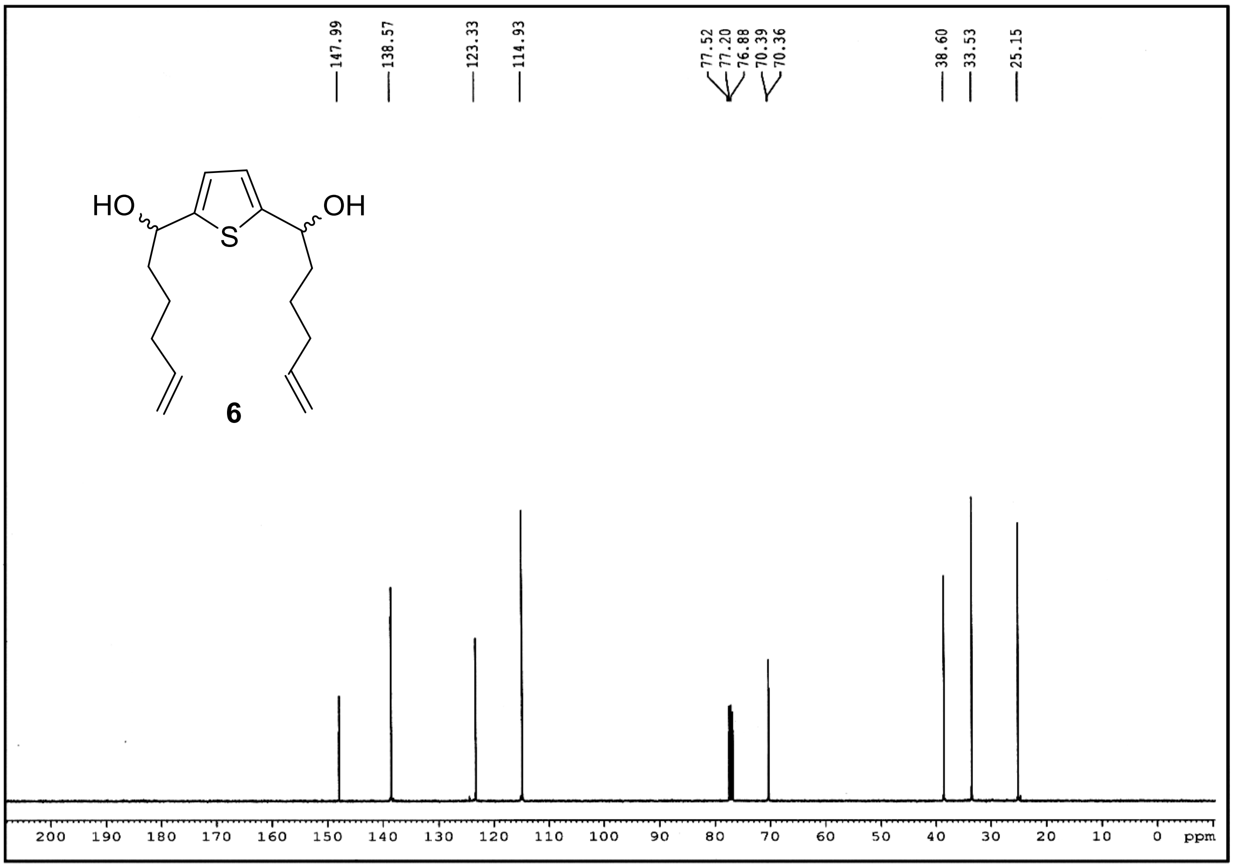

^{1}H NMR (400 MHz, $CDCl_3$) and ^{13}C NMR (100 MHz) of compound 6a

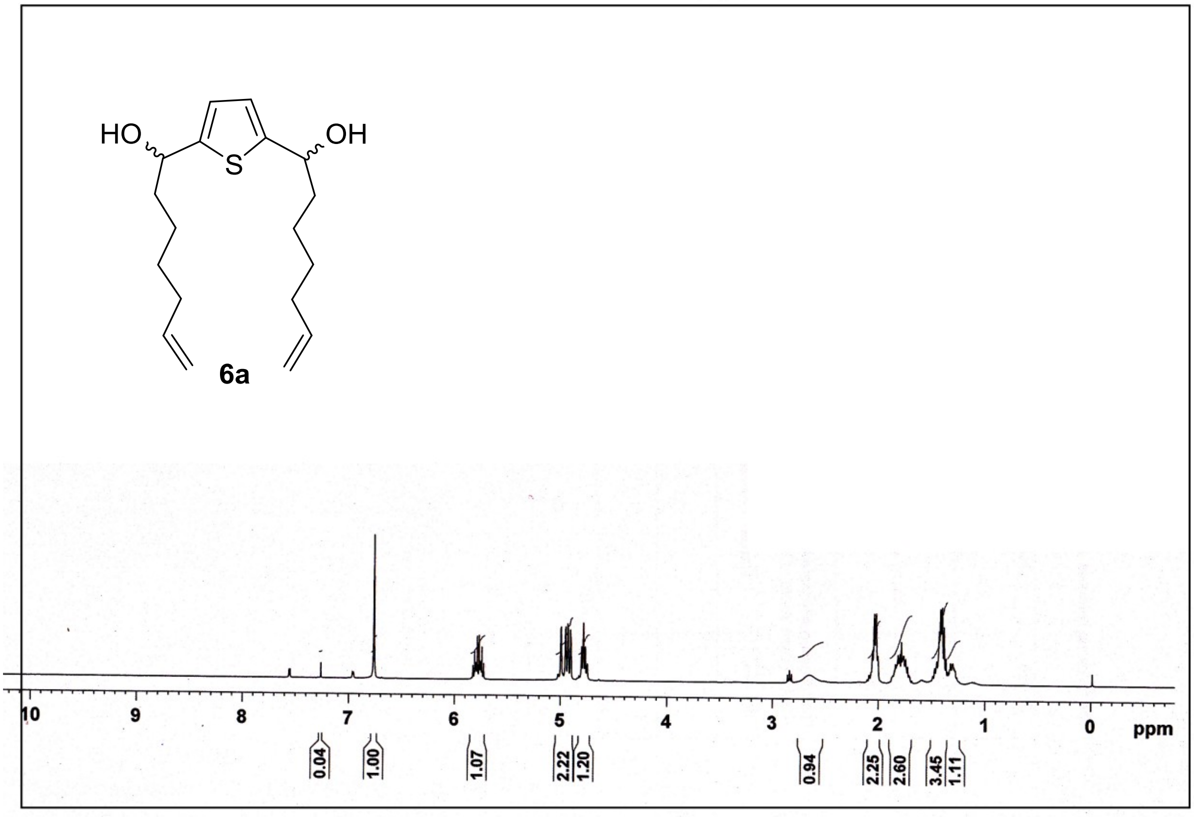

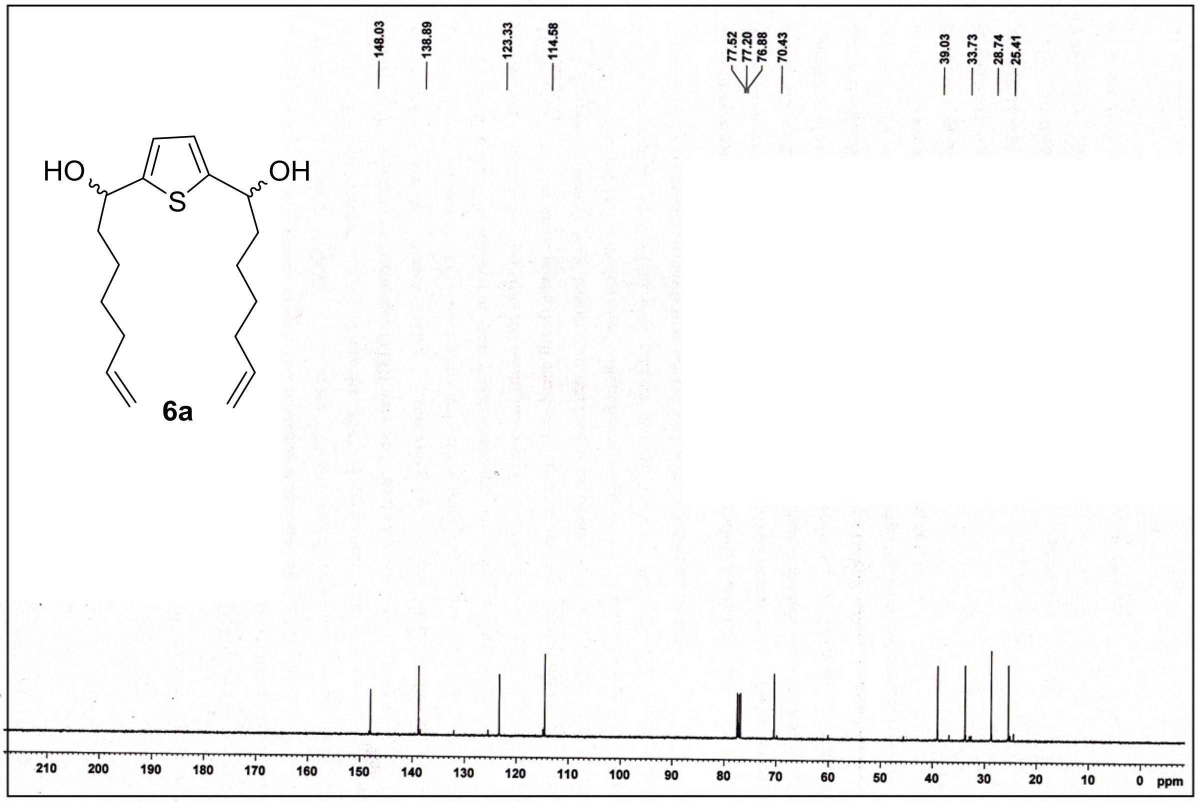

^{1}H NMR (400 MHz, $CDCl_3$) and ^{13}C NMR (100 MHz) of compound 3

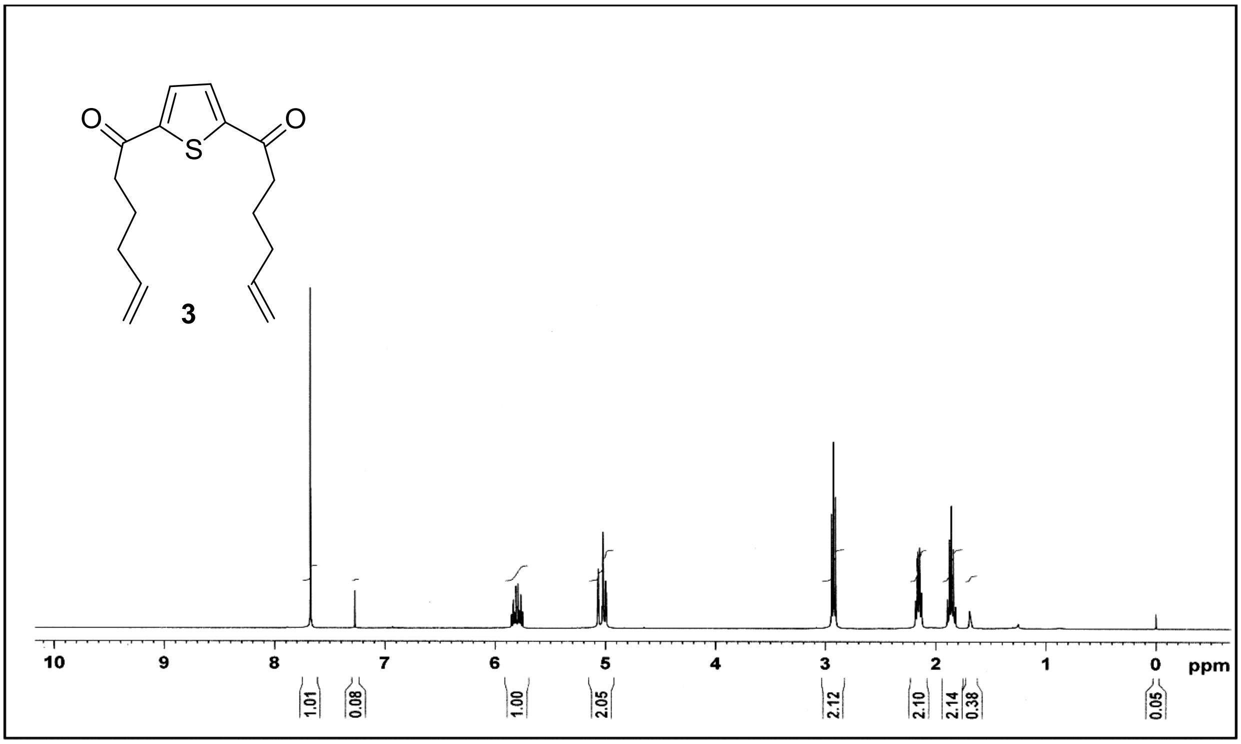

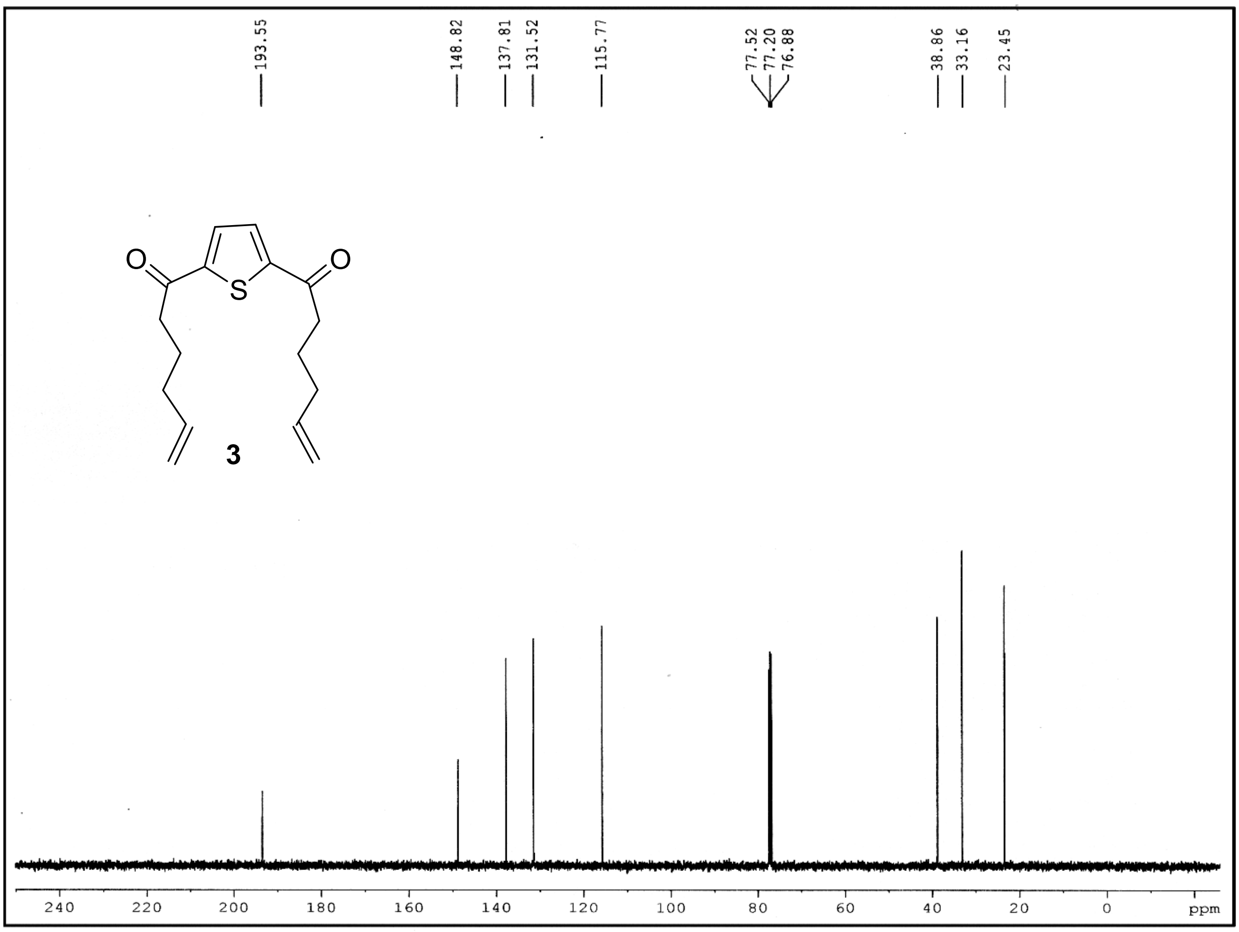

^{1}H NMR (400 MHz, $CDCl_3$) and ^{13}C NMR (100 MHz) of compound 3a

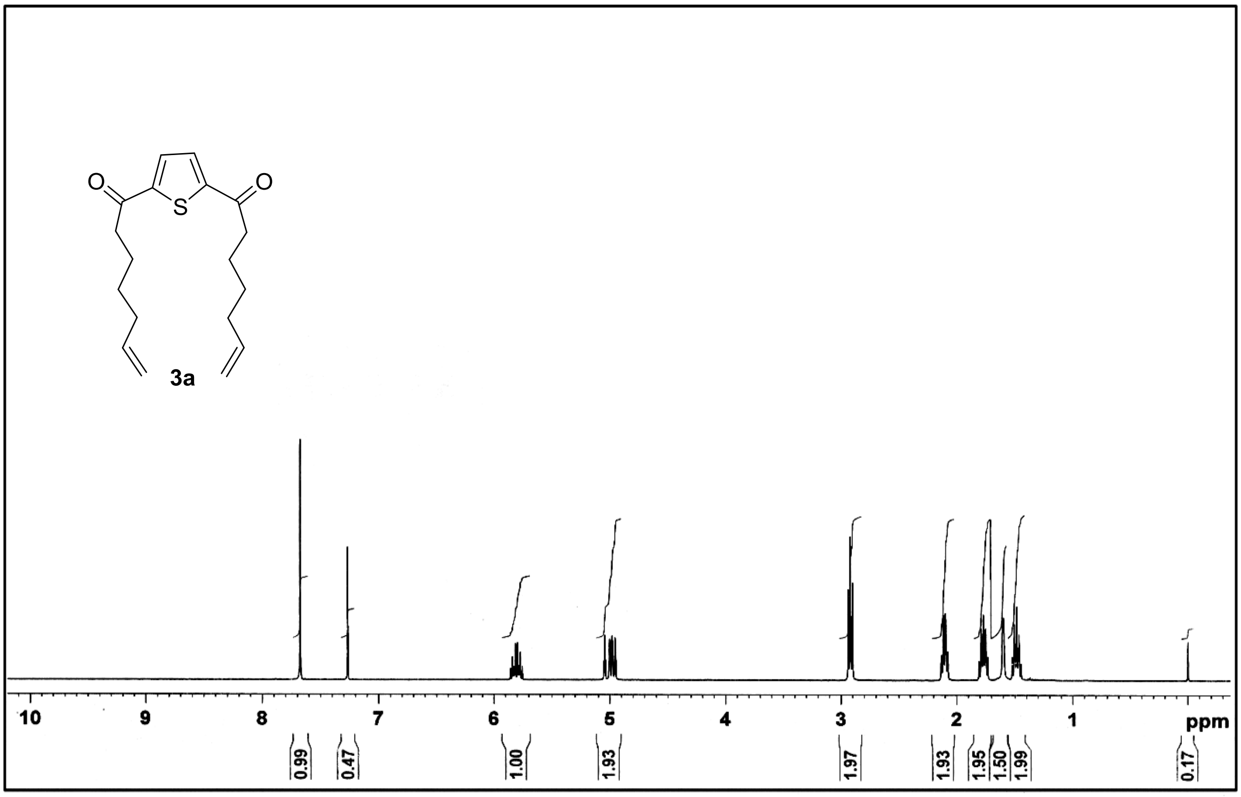

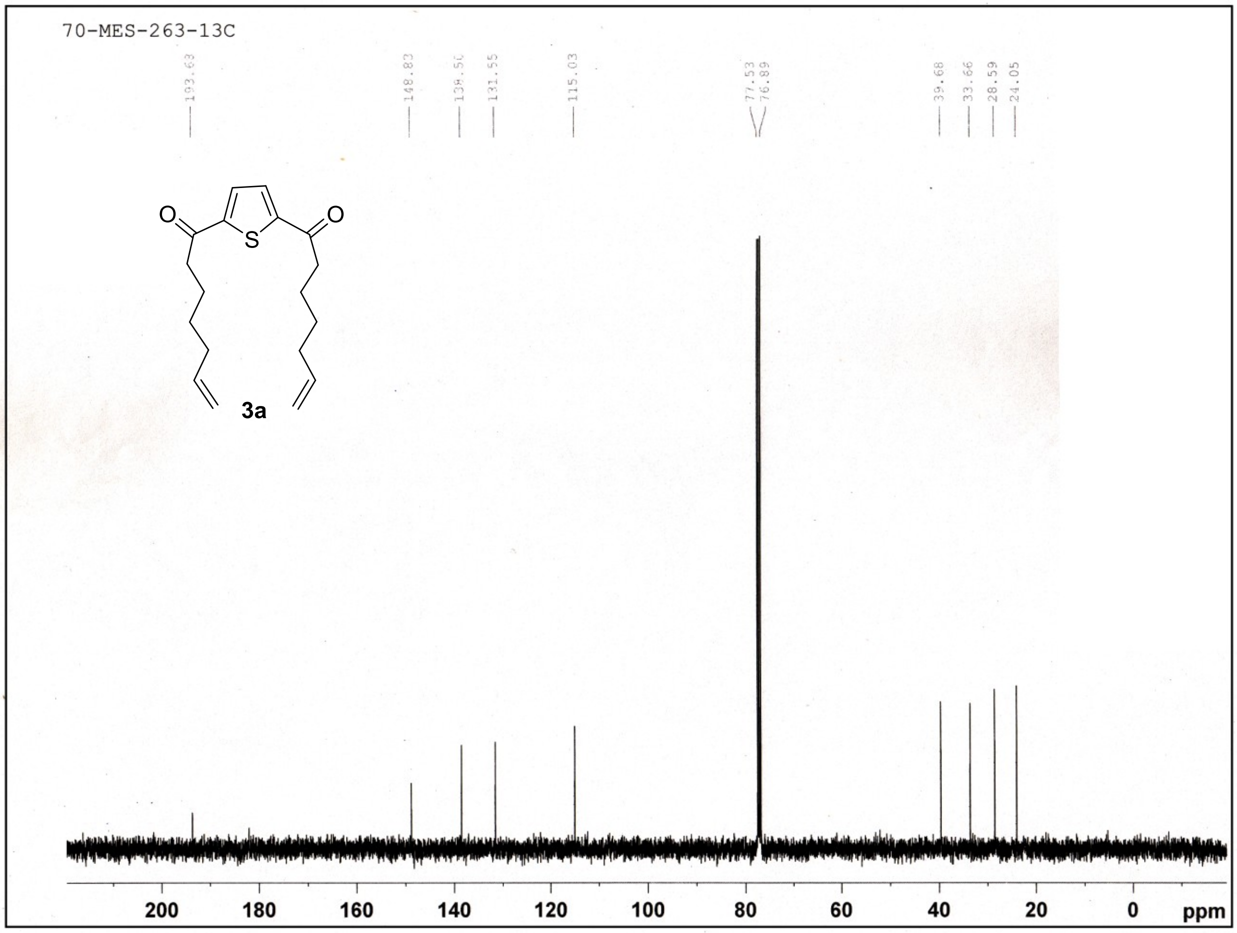

^{1}H NMR (500 MHz, $CDCl_3$) and ^{13}C NMR (100.6 MHz) of compound 5

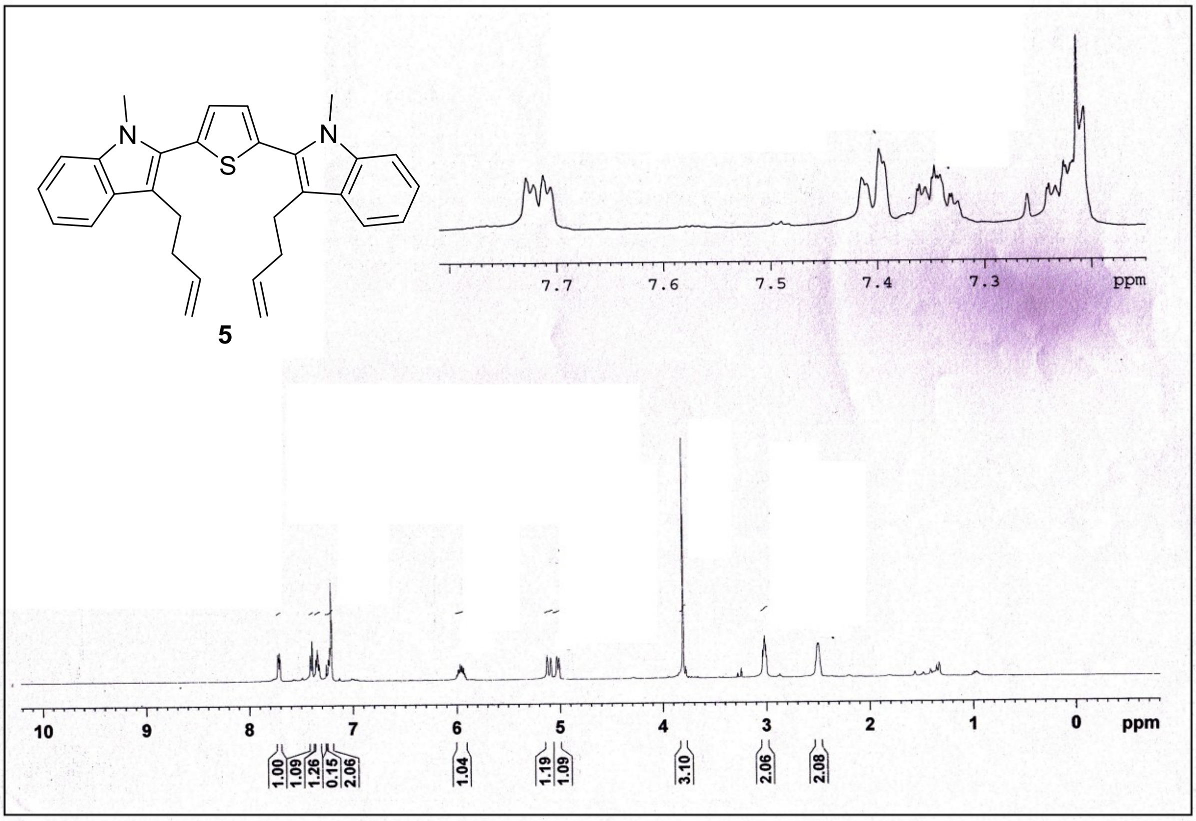

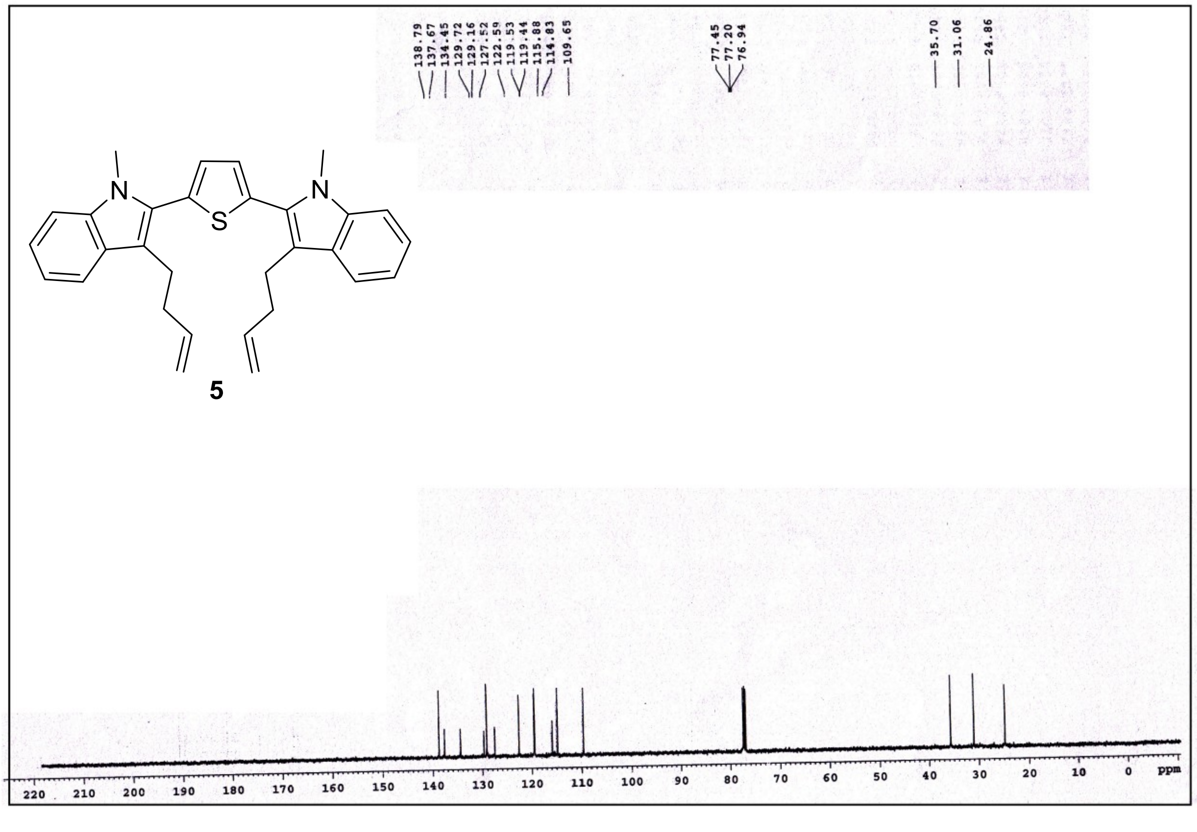

^{1}H NMR (400 MHz, $CDCl_3$) and ^{13}C NMR (125.6 MHz) of compound 1

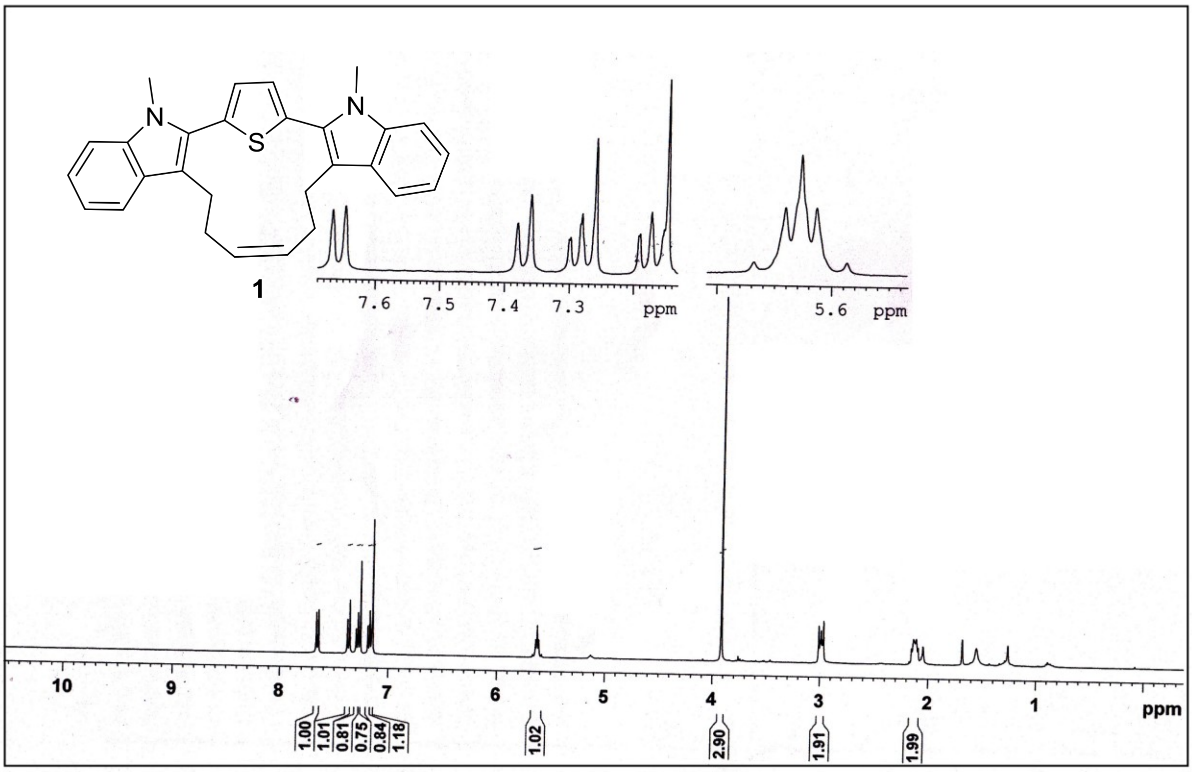

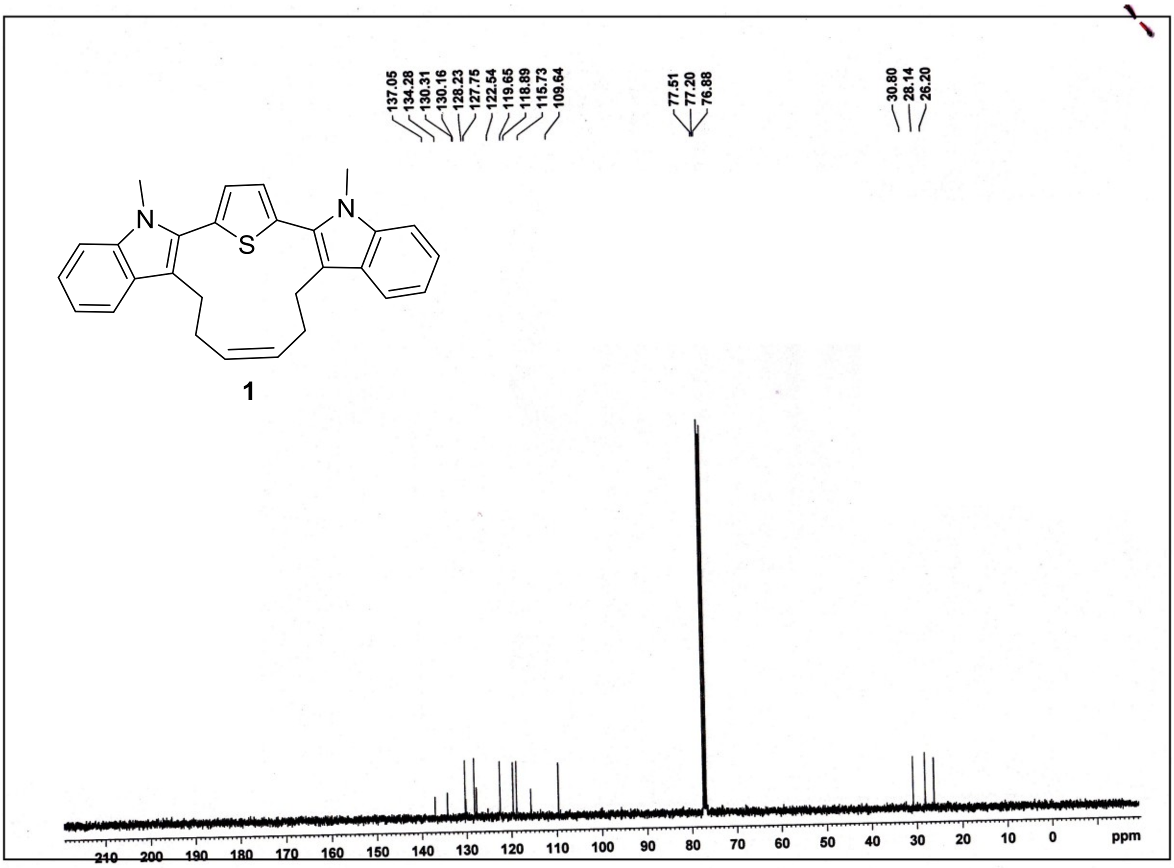

^{1}H NMR (500 MHz, $CDCl_3$) and ^{13}C NMR (125.6 MHz) of compound 5a

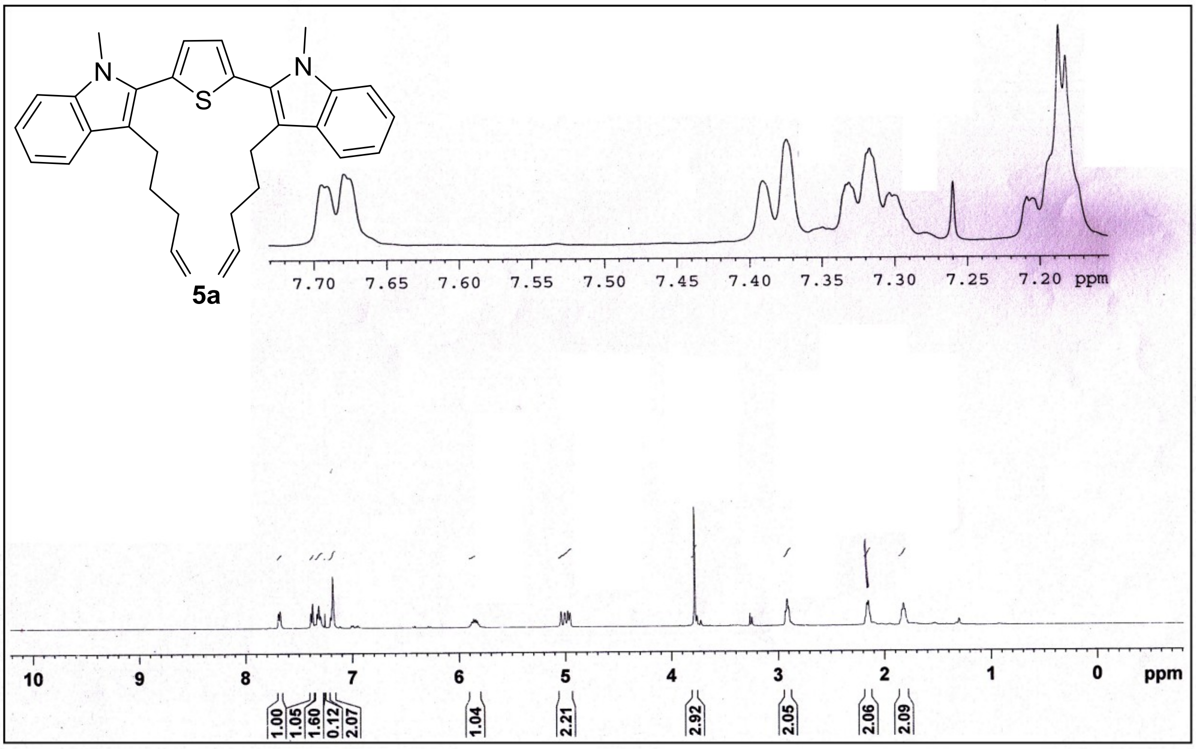

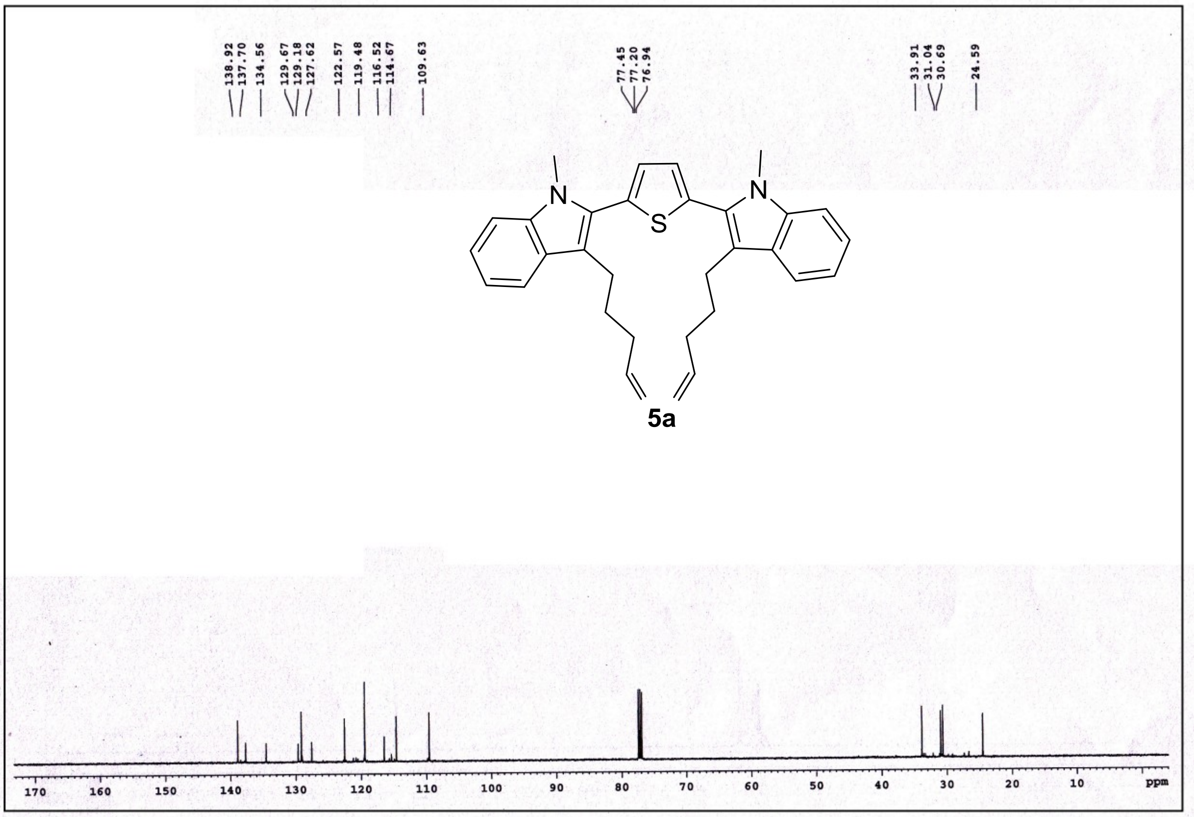

^{1}H NMR (500 MHz, $CDCl_3$) and ^{13}C NMR (125.6 MHz) of compound 1a

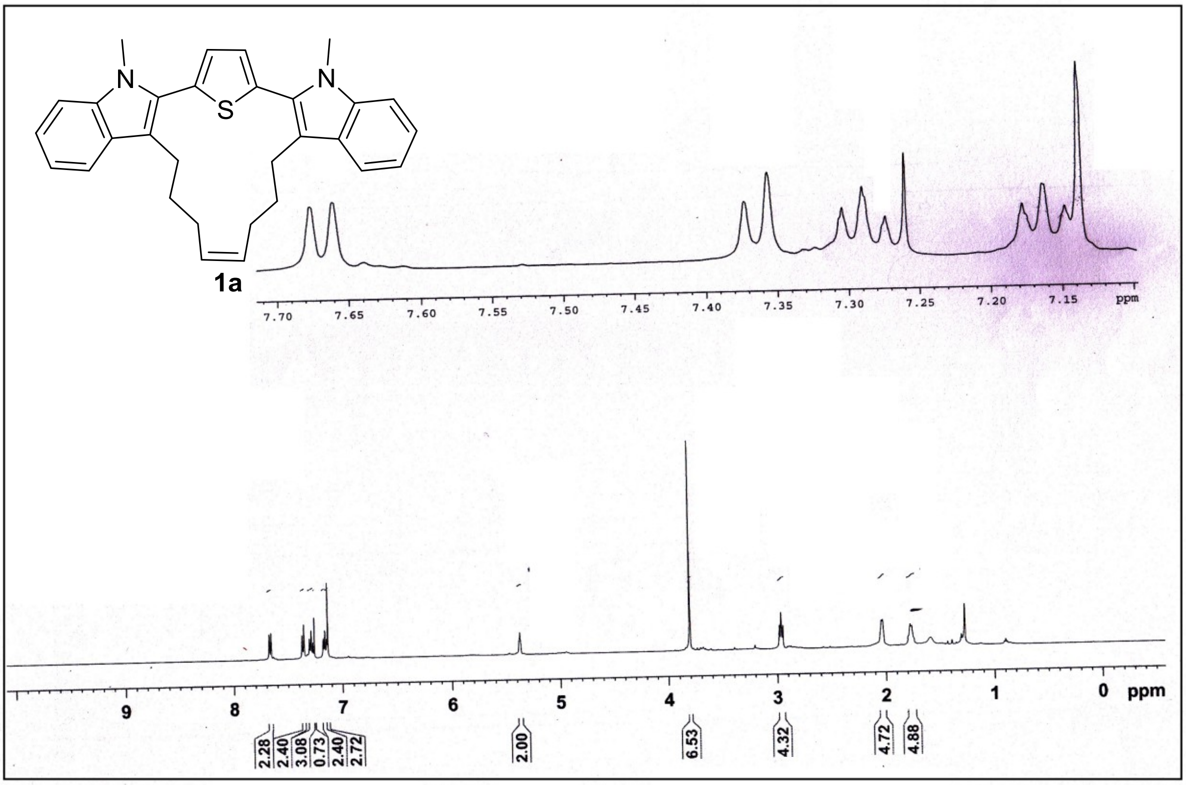

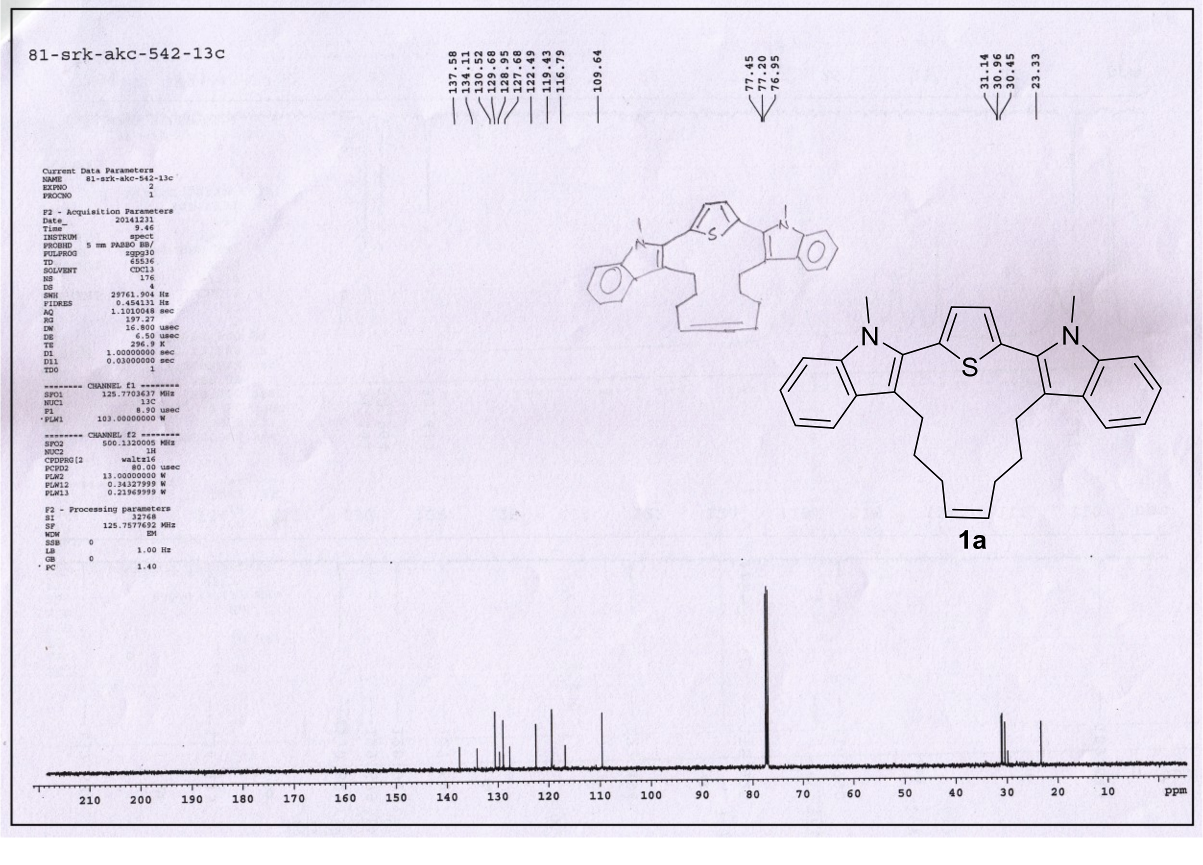

Supplement: File 1 — Copies of 1H, 13C NMR and HRMS spectra for all new compounds. [file Beilstein_J_Org_Chem-11-1514-s001.pdf]
